# Beyond surgery: clinical and economic impact of Enhanced Recovery After Surgery programs

**DOI:** 10.1186/s12913-018-3824-0

**Published:** 2018-12-29

**Authors:** Gaëtan-Romain Joliat, Olle Ljungqvist, Tracy Wasylak, Oliver Peters, Nicolas Demartines

**Affiliations:** 1 Department of Visceral Surgery, University Hospital CHUV, Rue du Bugnon 46, 1011 Lausanne, Switzerland; 2 Department of Surgery, Örebro University and University Hospital, Örebro, Sweden; 3 Alberta Health Services, Alberta, Canada; 4 Deputy Director General, University Hospital CHUV, Lausanne, Switzerland

**Keywords:** Enhanced recovery, Surgery, Implementation

## Abstract

**Background:**

Enhanced Recovery After Surgery (ERAS) is a perioperative management based on multimodality and multidisciplinary work. ERAS has been shown to have important clinical and economic benefits, but its spread remains slow worldwide.

**Discussion:**

This manuscript reviews the overall program benefits and focuses on important aspects for implementation well beyond surgery.

**Summary:**

Implementation of ERAS pathways improves clinical outcomes and induces substantial economic gains. ERAS is the current surgical revolution.

## Background

While surgeons mainly focus on techniques and technologies, new strategies that affect system performance offer many benefits for patients, healthcare providers, and health systems. Enhanced Recovery After Surgery (ERAS) is a systematized and validated perioperative management model based on best available evidence [1]. ERAS pathways decrease surgical stress, maintain physiological homeostasis, and improve postoperative recovery [1]. ERAS is a multidisciplinary management strategy encompassing several items and interventions, challenging old dogmas such as preoperative fasting [1]. The success of ERAS pathways depends on clinical leadership, site coordination, application of evidence-based care protocol items without any patient selection, and continuous audit of the outcomes and processes behind the performance [2]. The international ERAS® Society (www.erassociety.org) has published evidence-based guidelines for many surgical procedures. Implementation of these ERAS guidelines have been shown to substantially reduce postoperative complications, length of stay (LOS) and overall costs, and to increase both patient and staff satisfaction [3–5]. Two recent reports also suggested that ERAS implementation may be associated with improved long-term survival [2, 6].

There is limited documentation of the spread and scale of ERAS protocols across hospitals, stakeholders, and health systems, in a time when health systems are under increasing pressure for improved care at lower cost. This short article aims to analyze important clinical and economic outcomes of ERAS implementation to support healthcare providers and managers in their decision making to sustain and improve health care.

Due to changing demography and advancements in anesthesiology and in surgical strategies, hospitals will use a growing part of their bed capacity for complex multimorbid surgical patients. A strategy will therefore be needed to develop hospital capacity in a rational way. There is evidence showing that variation in care provision and outcomes is huge at almost every level – between countries, within countries, within hospitals, and often between individual doctors. The main reason for these discrepancies is that few evidence-based treatments are used and measured. Evidence-based standard processes should be defined and tracked to minimize unnecessary variation in care and allow timely escalation procedures in non-standard situations. The goal is optimal use of economic resources and clinical interventions to reach equitable and efficient care. ERAS fits in this global approach. It includes process standardization and measurement, as well as interdisciplinary and systematic improvement methods to put in place a sustainable system for continuous enhanced care. In addition, ERAS fulfills the recently described Quadruple Aim: achieving better patient outcomes, at lower cost, with improved patient, medical, nursing, and provider satisfaction [7].

### Impact of ERAS on morbidity and costs

The major positive consequence of implementing ERAS pathways is improvement of patient outcomes at decreased costs. Several studies suggest that ERAS decreased postoperative complications. In colorectal surgery, a meta-analysis of 16 randomized trials with 2376 patients showed decreased postoperative morbidity by 40% (RR = 0.6, 95% CI: 0.46–0.76) [3]. In pancreas surgery, a meta-analysis of 14 non-randomized studies also showed a diminution of overall morbidity (OR = 0.63, 95% CI: 0.54–0.74) [8]. In liver surgery, complications were decreased by 30 to 50% [4, 9]. It may be surprising that a sum of relatively simple perioperative measures such as early mobilization and oral alimentation impacts patient outcomes that much. This highlights the power of all ERAS items working together rather than single elements only and assessing their individual application. However, the physiologic and underlying mechanisms of ERAS remain debated and subject to investigations. Pathophysiology and improved immunity play a role, but standardization of clinical pathways probably has an important influence as well. Moreover, the decrease in complications is partly responsible for the decreased LOS observed in ERAS patients. Of note, faster hospital discharge with ERAS does not result in increased readmission rates [3].

In decreasing complications, ERAS protocols produced reduced LOS and costs. Several reports from Europe and North America measured ERAS cost savings. For example, in colorectal, pancreas, and liver surgery, a Swiss university hospital measured reductions of €1651, €7738, and €3080 per patient, respectively, accounting for almost $1 million USD cost savings for the 198 assessed patients in these three studies [5, 10, 11]. In Canada, implementation of ERAS across an entire provincial healthcare system in Alberta showed net cost savings ranging from $2806 to $5898 USD per patient in a cohort of consecutive colorectal patients [12]. Thus, ERAS was cost-beneficial and also cost-effective as complications were decreased. Cost savings in colorectal surgery were more substantial in Canada than in Switzerland in the mentioned studies. Overall complication and readmission rates were decreased in the Canadian study, whereas no difference was noted in the Swiss article. This can be a potential explanation for the observed difference in cost reduction. Moreover, in Canada, ERAS was implemented across an entire health care system (Alberta province) compared to Swiss data originating from one single center, which can induce more substantial cost savings globally. Finally, salary (e.g., ERAS nurse) and material costs are probably less expensive in Canada (Switzerland is a known expensive country), allowing cheaper specific ERAS costs of implementation in Canada. Even if primary investments are necessary, return on investment is substantial, reported at a factor of four [13]. As ERAS induces cost savings per patient, the more patients enrolled, the more savings. Important factors for ERAS long-term success are changes in management of care processes and time investment to form a multidisciplinary and multiprofessional ERAS team along with the use of dedicated protocols, continuous audit, and feedback [13].

### ERAS implementation

A barrier for ERAS implementation may be the reluctance of managers to invest in ERAS team building, database, and systematic audit [13]. A way to overcome the barriers faced for global ERAS implementation might be to increase objective data on financial benefits of ERAS, as shown in recent publications on ERAS costs. Another possibility could be to improve awareness of the advantages of an ERAS management (via advertisements or information campaigns) in the general population, which could convince the managers to invest in such successful programs. To diminish primary investments, ERAS implementation on multiple sites at the same time across an entire health care system or a specific geographical area could be realized with substantial lower costs. The real question to ask would be: what does a hospital lose by not applying ERAS? For example positive clinical and economic impact of expansive new surgical technologies has not been demonstrated yet. Investments in robotic surgery are made at a cost 20 times higher than investment in ERAS. It is surprising that departments that invested in new technologies did not invest in ERAS first in order to improve care and save money. Cost savings from ERAS implementation could then as a result be used to invest in new technologies. In fact, cost savings possibly induced by new technologies remain to be unequivocally demonstrated [14, 15]. Technology advancements are of course important in surgery, but perioperative management goes far beyond surgical technique in terms of impact on outcomes and costs and can be introduced with lower investment. Moreover, chairs of surgical departments might be hostile to ERAS implementation because it would mean important changes in their clinical habits and established management pathways. Another obstacle may also be the fact that many centers are claiming to apply ERAS already, but without controlled application of protocols and ERAS pathways.

It has been demonstrated that ERAS positively influences patient subjective well-being with better health-related quality of life, less pain or fatigue, and better patient satisfaction [16, 17]. ERAS puts the patient in the center of its perioperative management and recovery team and empowers him or her by increasing motivation to recover quickly and accepting responsibilities in their own management and recovery plan. In major gynecologic surgery, a before-and-after ERAS implementation study reported significantly better pain control and better patient information [18]. Patient information and education, as well as increased communication are often neglected key factors in the surgical process.

ERAS is also well accepted and supported by medical and nursing staff. Some prospective data from Switzerland suggested that overall nursing workload was decreased after ERAS implementation [19]. Workload was assessed by a standardized and validated point system (PRN) and decreased by about 15% [19]. Furthermore, higher compliance to ERAS protocols was associated with reduced nursing workload [19]. In another report, paramedical staff described ERAS as a successful change without compromising workload and work environment of the nursing staff, confirming good general appraisal of ERAS by care providers [20].

## Conclusions

With health systems worldwide looking at sustainable ways to achieve the above mentioned Quadruple Aim, ERAS provides an evidence-based solution for surgical patients [7]. This surgical transformation significantly improves system performances both clinically and financially. The concept has been proven for almost every major operation, and ERAS implementation programs have been successfully implemented in more than 30 countries around the world [13]. ERAS is not a pilot project anymore with more than 35,000 patients included worldwide in late 2017.

Therefore, why does ERAS not spread faster? Perhaps is ERAS wrongly viewed as improvement for selected patients and specific operations? In fact ERAS is a means to transform perioperative management for all surgical patients without any selection within an entire hospital. In addition the implementation needs initial investments with a systematic implementation course instead of simple user notice and time to implement and run ERAS with dedicated resources to support the changes at a unit and site level. This may be perceived negatively because cost savings are not necessarily achieved in the same budgets where the changes are made. In some units there is also a lack of clinical leadership to change for ERAS. Implementation needs work, time, and investment, and ERAS is not magic, but just the systematic application of an evidence-based protocol that drives surgical transformation. It is necessary that boardrooms where investment decisions are made understand the process, support clinicians to make the required changes that repeatedly demonstrated the overwhelming clinical and financial benefits, and include ERAS in the hospital business plan (Table 1). Moreover, sustainability and viability of ERAS should be taken into consideration (since savings are made for every enrolled patient). However, to enjoy the financial gains across a hospital setting, making spread and scale of ERAS protocols an expectation for every hospital will facilitate the gains more quickly. In the future, expansion of ERAS in low- and middle-income countries might be challenging, because investments are difficult to obtain. In addition, implementation of ERAS across entire health care systems will be a demanding but necessary task to develop ERAS internationally. Finally, ERAS pathway has to be adaptable to local conditions in various countries and evolves over time as the scientific evidence grows. ERAS is the current surgical revolution to improve clinical outcomes and economic efficiency in health care systems, far beyond surgical techniques and technologies. The main questions remaining are – what are managers and leaders waiting for? And how long will patients still accept treatment at a non-ERAS hospital?

**Table 1:** Synoptic table of the decrease in complications and costs after ERAS implementation in various types of surgery (adapted from cited references)

ERAS	Complication decrease (%)	Cost decrease per patient (Euros)
Colorectal surgery	40–50% [3, 21]	€1651-5577 [11, 12]
Pancreas surgery	14–37% [5, 8]	€325–7738 [5, 22]
Liver surgery	30–50% [4, 9]	€3080 [10]
Gastric surgery	0–10% [23, 24]	€478–514 [25, 26]
Gynecologic surgery	0–19% [18, 27]	€1202-7187 [18, 27]
Urologic surgery	0–15% [28, 29]	N/A

*N/A*: not available

## Abbreviations

ERAS: Enhanced Recovery After Surgery
LOS: Length of stay
